# Alcoholism and the Brain: An Overview

**Published:** 2003

**Authors:** Marlene Oscar-Berman, Ksenija Marinkovic

**Affiliations:** Marlene Oscar-Berman, Ph.D., is a professor in the Departments of Anatomy and Neurobiology, Psychiatry, and Neurology, Boston University School of Medicine, and a research career scientist at the U.S. Department of Veterans Affairs Healthcare System, Jamaica Plain Division, Boston, Massachusetts. Ksenija Marinkovic, Ph.D., is a research scientist at the Athinoula A. Martinos Center for Biomedical Imaging, instructor in the Radiology Department at Harvard Medical School, and assistant in Neuroscience at the Massachusetts General Hospital, Boston, Massachusetts

**Keywords:** neurobehavioral theory of AODU (alcohol and other drug use), alcoholic brain syndrome, brain atrophy, neuropsychological assessment, neurotransmission, risk factors, comorbidity, disease susceptibility, neuroimaging, treatment factors, survey of research

## Abstract

Alcoholism can affect the brain and behavior in a variety of ways, and multiple factors can influence these effects. A person’s susceptibility to alcoholism-related brain damage may be associated with his or her age, gender, drinking history, and nutrition, as well as with the vulnerability of specific brain regions. Investigators use a variety of methods to study alcoholism-related brain damage, including examining brains of deceased patients as well as neuroimaging, a technique that enables researchers to test and observe the living brain and to evaluate structural damage in the brain.

The brain, like most body organs, is vulnerable to injury from alcohol consumption. The risk of brain damage and related neurobehavioral deficits varies from person to person. This article reviews the many factors that influence this risk, the techniques used to study the effects of alcoholism^1^ on the brain and behavior, and the implications of this research for treatment.

About half of the nearly 20 million alcoholics in the United States seem to be free of cognitive impairments. In the remaining half, however, neuropsychological difficulties can range from mild to severe. For example, up to 2 million alcoholics develop permanent and debilitating conditions that require lifetime custodial care (Rourke and Löberg 1996). Examples of such conditions include alcohol-induced persisting amnesic disorder (also called Wernicke-Korsakoff syndrome) and dementia, which seriously affects many mental functions in addition to memory (e.g., language, reasoning, and problem-solving abilities) (Rourke and Löberg 1996). Most alcoholics with neuropsychological impairments show at least some improvement in brain structure and functioning within a year of abstinence, but some people take much longer (Bates et al. 2002; Gansler et al. 2000; Sullivan et al. 2000). Unfortunately, little is known about the rate and extent to which people recover specific structural and functional processes after they stop drinking. However, research has helped define the various factors that influence a person’s risk for experiencing alcoholism-related brain deficits, as the following sections describe.

## Risk Factors and Comorbid Conditions That Influence Alcohol-Related Brain Damage

Alcoholism’s effects on the brain are diverse and are influenced by a wide range of variables (Parsons 1996). These include the amount of alcohol consumed, the age at which the person began drinking, and the duration of drinking; the patient’s age, level of education, gender, genetic background, and family history of alcoholism; and neuropsychiatric risk factors such as alcohol exposure before birth and general health status. Overall physical and mental health is an important factor because comorbid medical, neurological, and psychiatric conditions can interact to aggravate alcoholism’s effects on the brain and behavior. Examples of common comorbid conditions include:

- Medical conditions such as malnutrition and diseases of the liver and the cardiovascular system
- Neurological conditions such as head injury, inflammation of the brain (i.e., encephalopathy), and fetal alcohol syndrome (or fetal alcohol effects)
- Psychiatric conditions such as depression, anxiety, post-traumatic stress disorder, schizophrenia, and the use of other drugs (Petrakis et al. 2002).

These conditions also can contribute to further drinking.

## Models for Explaining Alcohol-Related Brain Damage

Some of the previously mentioned factors that are thought to influence how alcoholism affects the brain and behavior have been developed into specific models or hypotheses to explain the variability in alcoholism-related brain deficits. The accompanying table lists the prevailing models (Oscar-Berman 2000). It should be noted that the models that focus on individual characteristics cannot be totally separated from models that emphasize affected brain systems because all of these factors are interrelated. Several of the models have been evaluated using specialized tests that enable researchers to make inferences about the type and extent of brain abnormalities.

**Table:** Hypotheses Proposed to Explain the Consequences of Alcoholism for the Brain

*Hypotheses Emphasizing the Personal Characteristics Associated With Vulnerability*	
Characteristic	Hypothesis
Aging	Premature aging hypothesis: Alcoholism accelerates aging. Brains of alcoholics resemble brains of chronologically old nonalcoholics. This may occur at the onset of problem drinking (“accelerated aging”) or later in life when brains are more vulnerable (“increased vulnerability” or “cumulative effects”).
Gender	Alcoholism affects women more than men. Although women and men metabolize alcohol differently, it is not yet clear if women’s brains are more vulnerable than men’s brains to the effects of alcoholism.
Family history	Alcoholism runs in families; thus, children of alcoholics face increased risk of alcoholism and associated brain changes.
Vitamin deficiency	Thiamine deficiency can contribute to damage deep within the brain, leading to severe cognitive deficits.

***Hypotheses Emphasizing the Vulnerability of Brain Regions or Systems***	
**Region/System**	**Hypothesis**

Entire brain	Vulnerable to cerebral atrophy.
Limbic system, thalamus, and hypothalamus	Vulnerable to alcohol-induced persisting amnesic disorder (also known as Wernicke-Korsakoff syndrome).
Frontal lobe systems	More vulnerable to the effects of alcoholism than other brain regions/systems.
Right hemisphere	More vulnerable to the effects of alcoholism than the left hemisphere.*
Neurotransmitter systems (e.g., gamma-aminobutyric acid (GABA), glutamate, dopamine, acetylcholine, and serotonin systems)	Several neurotransmitter systems are vulnerable to effects of alcoholism.

* The right hemisphere is also believed to be more vulnerable to the effects of normal aging than the left hemisphere, which is taken as support for the premature aging hypothesis listed above.

NOTE: These hypotheses are not mutually exclusive; some are interrelated. Supporting data for these models come from neurobehavioral and electrophysiological studies, brain scans, and post mortem neuropathology.

### Models Based on Characteristics of Individual Alcoholics

#### Premature Aging Hypothesis

According to this hypothesis, alcoholism accelerates natural chronological aging, beginning with the onset of problem drinking.

An alternate version suggests that older patients (age 50 and older) are especially susceptible to the cumulative effects of alcoholism, and aging is accelerated only later in life. The preponderance of scientific evidence suggests that although alcoholism-related brain changes may mimic some of the changes seen in older people, alcoholism does not cause premature aging. Rather, the effects of alcoholism are disproportionately expressed in older alcoholics (Oscar-Berman 2000).

#### Gender

Although it has been hypothesized that women’s brain functioning is more vulnerable to alcoholism than men’s, studies of gender differences have not consistently found this to be true (see Wuethrich 2001 for a review), even though women and men metabolize alcohol differently (i.e., women achieve higher blood alcohol contents [BACs] than men after consuming the same amount of alcohol). However, it is not known whether this comparison between men and women holds among older populations (Oscar-Berman 2000).

#### Family History

Family history of alcoholism has been found to be important because it can influence such things as tolerance for alcohol and the amount of consumption needed to feel alcohol’s effects. Also, studies examining brain functioning in people with and without a positive family history of alcoholism have shown that there are clear differences between the groups on measures of brain electrical activity (Porjesz and Begleiter 1998).

#### Vitamin Deficiency

Research on malnutrition, a common consequence of poor dietary habits in some alcoholics, indicates that thiamine deficiency (vitamin B_1_) can contribute to damage deep within the brain, leading to severe cognitive deficits (Oscar-Berman 2000). The exact location of the affected parts of the brain and underlying neuropathological mechanisms are still being researched (see the next section).

### Models Based on Vulnerable Brain Systems

**Figure 1:**
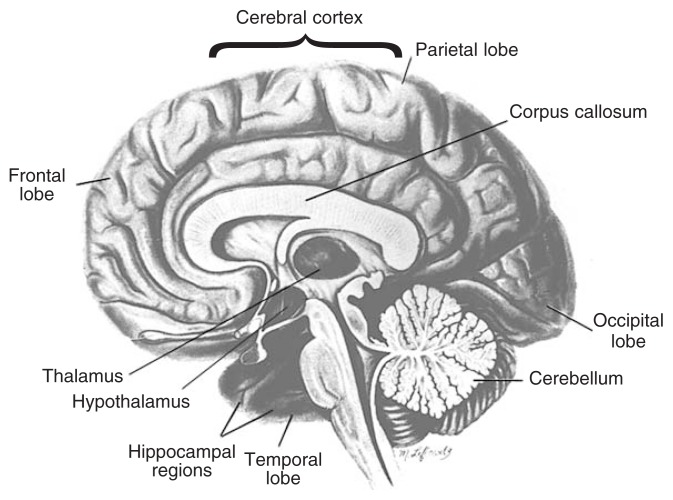
Schematic drawing of the human brain, showing regions vulnerable to alcoholism-related abnormalities.

The outer, convoluted layer of brain tissue, called the cerebral cortex or the gray matter, controls most complex mental activities (see figure 1). Just beneath it are the nerve fibers, called the white matter, that connect different cortical regions and link cortical cells with other structures deep inside the brain (subcortical regions).

Areas of the brain that are especially vulnerable to alcoholism-related damage are the cerebral cortex and subcortical areas such as the limbic system (important for feeling and expressing emotions), the thalamus (important for communication within the brain), the hypothalamus (which releases hormones in response to stress and other stimuli and is involved in basic behavioral and physiological functions), and the basal forebrain (the lower area of the front part of the brain, involved in learning and memory) (Oscar-Berman 2000). Another brain structure that has recently been implicated is the cerebellum (Sullivan 2000), situated at the base of the brain, which plays a role in posture and motor coordination and in learning simple tasks.

#### Alcohol-Related Brain Atrophy

According to one hypothesis, shrinkage (i.e., atrophy) of the cerebral cortex and white matter, as well as possible atrophy of basal forebrain regions, may result from the neurotoxic effects of alcohol (Lishman 1990). Furthermore, thiamine deficiency may result in damage to portions of the hypothalamus (perhaps because blood vessels break in that region). According to this hypothesis, alcoholics who are susceptible to alcohol toxicity^2^ may develop permanent or transient cognitive deficits associated with brain shrinkage. Those who are susceptible to thiamine deficiency will develop a mild or transient amnesic disorder, with short-term memory loss as the salient feature. Patients with dual vulnerability, those with a combination of alcohol neurotoxicity and thiamine deficiency, will have widespread damage to large regions of the brain, including structures deep within the brain such as the limbic system. These people will exhibit severe short-term memory loss and collateral cognitive impairments (Oscar-Berman 2000).

#### Frontal Lobe Vulnerability

Although alcoholics have diffuse damage in the cerebral cortex of both hemispheres of the brain, neuropathological studies performed on the brains of deceased patients as well as findings derived from neuroimaging studies of living brains point to increased susceptibility of frontal brain systems to alcoholism-related damage (Moselhy et al. 2001; Oscar-Berman 2000; Sullivan 2000). The frontal lobes are connected with all other lobes of the brain (i.e., the parietal, temporal, and occipital lobes on both halves of the brain; see figure 1), and they receive and send fibers to numerous subcortical structures. Behavioral neuroscientists have determined that the anterior region of the frontal lobes (i.e., the prefrontal cortex) is important for engaging in ordinary cognitive, emotional, and interpersonal activities. The prefrontal cortex is considered the brain’s executive— that is, it is necessary for planning and regulating behavior, inhibiting the occurrence of unnecessary or unwanted behaviors, and supporting adaptive “executive control” skills such as goal-directed behaviors, good judgment, and problem-solving abilities. Disruptions of the normal inhibitory functions of prefrontal networks often have the interesting effect of releasing previously inhibited behaviors. As a result, a person may behave impulsively and inappropriately, which may contribute to excessive drinking.

**Figure 2:**
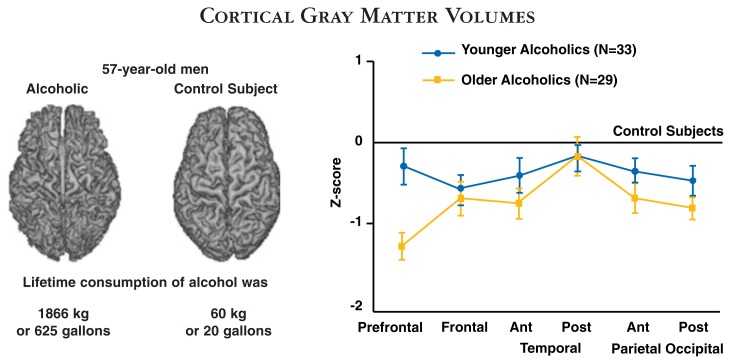
Brain MRI scans of age-equivalent men with different histories of alcohol use. The image shows clear evidence of brain shrinkage in the alcoholic compared with the control subject. The graph on the right shows that older alcoholics have less cortical tissue than younger alcoholics, and that the prefrontal cortex is especially vulnerable to alcohol’s effects. The location of the temporal, parietal, and occipital regions of the brain can be seen in figure 1. *Z-score is a mathematical measure that is useful for showing the difference between the recorded value and a “normal” value. SOURCE: Pfefferbaum et al. 1997.

There is evidence that the frontal lobes are particularly vulnerable to alcoholism-related damage, and the brain changes in these areas are most prominent as alcoholics age (Oscar-Berman 2000; Pfefferbaum et al. 1997; Sullivan 2000) (see figure 2). Other studies of frontal lobe function in older alcoholics have confirmed reports of a correlation between impaired neuropsychological performance (e.g., executive control skills, as noted above) and decreased blood flow or metabolism (energy use) in the frontal lobes, as seen using neuroimaging techniques (Adams et al. 1998).

#### Vulnerability of the Right Hemisphere

Some investigators have hypothesized that functions controlled by the brain’s right hemisphere are more vulnerable to alcoholism-related damage than those carried out by the left hemisphere (see Oscar-Berman and Schendan 2000 for review). Each hemisphere of the human brain is important for mediating different functions. The left hemisphere has a dominant role in communication and in understanding the spoken and written word. The right hemisphere is mainly involved in coordinating interactions with the three-dimensional world (e.g., spatial cognition).

Differences between the two cerebral hemispheres can easily be seen in patients with damage to one hemisphere but not the other (from stroke, trauma, or tumor). Patients with left hemispheric damage often have problems with language; patients with right hemispheric damage often have difficulty with maps, designs, music, and other nonlinguistic materials, and they may show emotional apathy.

Alcoholics may seem emotionally “flat” (i.e., they are less reactive to emotionally charged situations), and may have difficulty with the same kinds of tasks that patients with damage to the right hemisphere have difficulty with. New research has shown that alcoholics are impaired in emotional processing, such as interpreting nonverbal emotional cues and recognizing facial expressions of emotion (Kornreich et al. 2002; Monnot et al. 2002; Oscar-Berman 2000). Yet, despite the fact that emotional functioning can be similar in some alcoholics and people with right hemisphere damage, research provides only equivocal support for the hypothesis that alcoholism affects the functioning of the right hemisphere more than the left (Oscar-Berman and Schendan 2000). Impairments in emotional functioning that affect alcoholics may reflect abnormalities in other brain regions which also influence emotional processing, such as the limbic system and the frontal lobes.

#### Disruption of Neurotransmitter Systems

Brain cells (i.e., neurons) communicate using specific chemicals called neurotransmitters. Neuronal communication takes place at the synapse, where cells make contact. Specialized synaptic receptors on the surface of neurons are sensitive to specific neurotransmitters. Alcohol can change the activity of neurotransmitters and cause neurons to respond (excitation) or to interfere with responding (inhibition) (Weiss and Porrino 2002), and different amounts of alcohol can affect the functioning of different neurotransmitters. Over periods of days and weeks, receptors adjust to chemical and environmental circumstances, such as the changes that occur with chronic alcohol consumption, and imbalances in the action of neurotransmitters can result in seizures, sedation, depression, agitation, and other mood and behavior disorders.

The major excitatory neurotransmitter in the human brain is the amino acid glutamate. Small amounts of alcohol have been shown to interfere with glutamate action. This interference could affect several brain functions, including memory, and it may account for the short-lived condition referred to as “alcoholic blackout.” Chronic alcohol consumption increases glutamate receptor sites in the hippocampus, an area in the limbic system that is crucial to memory and often involved in epileptic seizures. During alcohol withdrawal, glutamate receptors that have adapted to the long-term presence of alcohol may become overactive, and this overactivity has been repeatedly linked to neuronal death, which is manifested by conditions such as stroke and seizures. Deficiencies of thiamine caused by malnutrition may contribute to this potentially destructive overactivity (Crews 2000).

Gamma-aminobutyric acid (GABA) is the major inhibitory neurotransmitter. Available evidence suggests that alcohol^3^ initially potentiates GABA’s effects (i.e., it increases inhibition, and often the brain becomes mildly sedated). However, over time, prolonged, excessive alcohol consumption reduces the number of GABA receptors. When the person stops drinking, decreased inhibition combined with a deficiency of GABA receptors may contribute to overexcitation throughout the brain. This in turn can contribute to withdrawal seizures within a day or two. It should be noted that the balance between the inhibitory action of GABA and the excitatory action of glutamate is a major determinant of the level of activity in certain regions of the brain; the effects of GABA and glutamate on withdrawal and brain function are probably interactive (see Valenzuela 1997 for review).

Alcohol directly stimulates release of the neurotransmitter serotonin, which is important in emotional expression, and of the endorphins, natural substances related to opioids, which may contribute to the “high” of intoxication and the craving to drink. Alcohol also leads to increases in the release of dopamine (DA), a neurotransmitter that plays a role in motivation and in the rewarding effects of alcohol (Weiss and Porrino 2002). Changes in other neurotransmitters such as acetylcholine have been less consistently defined. Future research should help to clarify the importance of many neurochemical effects of alcohol consumption. Furthermore, areas amenable to pharmacological treatment could be identified by studying regionally specific brain neurochemistry in vivo using neuroimaging methods such as positron emission tomography (PET) and single photon emission computerized tomography (SPECT) (described below). New information from neuroimaging studies could link cellular changes directly to brain consequences observed clinically. In the absence of a cure for alcoholism, a detailed understanding of the actions of alcohol on nerve cells may help in designing effective therapies.

## Techniques for Studying Alcohol-Related Brain Damage

Researchers use multiple methods to understand the etiologies and mechanisms of brain damage across subgroups of alcoholics. Behavioral neuroscience offers excellent techniques for sensitively assessing distinct cognitive and emotional functions—for example, the measures of brain laterality (e.g., spatial cognition) and frontal system integrity (e.g., executive control skills) mentioned earlier. Followup post mortem examinations of brains of well-studied alcoholic patients offer clues about the locus and extent of pathology and about neurotransmitter abnormalities. Neuroimaging techniques provide a window on the active brain and a glimpse at regions with structural damage.

### Behavioral Neuroscience

Behavioral neuroscience studies the relationship between the brain and its functions—for example, how the brain controls executive functions and spatial cognition in healthy people, and how diseases like alcoholism can alter the normal course of events. This is accomplished by using specialized tests designed expressly to measure the functions of interest. Among the tests used by scientists to determine the effects of alcoholism on executive functions controlled by the frontal lobes are those that measure problem-solving abilities, reasoning, and the ability to inhibit responses that are irrelevant or inappropriate (Moselhy et al. 2001; Oscar-Berman 2000). Tests to measure spatial cognition controlled by the right hemisphere include those that measure skills important for recognizing faces, as well as those that rely on skills required for reading maps and negotiating two- and three-dimensional space (visuospatial tasks) (Oscar-Berman and Schendan 2000). With the advent of sophisticated neuroimaging techniques (described below), scientists can even observe the brain while people perform many tasks sensitive to the workings of certain areas of the brain.

### Neuropathology

Researchers have gained important insights into the anatomical effects of long-term alcohol use from studying the brains of deceased alcoholic patients. These studies have documented alcoholism-related atrophy throughout the brain and particularly in the frontal lobes (Harper 1998). Post mortem studies will continue to help researchers understand the basic mechanisms of alcohol-induced brain damage and regionally specific effects of alcohol at the cellular level.

### Neuroimaging

Remarkable developments in neuroimaging techniques have made it possible to study anatomical, functional, and biochemical changes in the brain that are caused by chronic alcohol use. Because of their precision and versatility, these techniques are invaluable for studying the extent and the dynamics of brain damage induced by heavy drinking. Because a patient’s brain can be scanned on repeated occasions, clinicians and researchers are able to track a person’s improvement with abstinence and deterioration with continued abuse. Furthermore, brain changes can be correlated with neuropsychological and behavioral measures taken at the same time. Brain imaging can aid in identifying factors unique to the individual which affect that person’s susceptibility to the effects of heavy drinking and risk for developing dependence, as well as factors that contribute to treatment efficacy.

#### Imaging of Brain Structure

With neuroimaging techniques such as computerized tomography (CT) and magnetic resonance imaging (MRI), which allow brain structures to be viewed inside the skull, researchers can study brain anatomy in living patients. CT scans rely on x-ray beams passing through different types of tissue in the body at different angles. Pictures of the “inner structure” of the brain are based on computerized reconstruction of the paths and relative strength of the x-ray beams. CT scans of alcoholics have revealed diffuse atrophy of brain tissue, with the frontal lobes showing the earliest and most extensive shrinkage (Cala and Mastaglia 1981).

MRI techniques have greatly influenced the field of brain imaging because they allow noninvasive measurement of both the anatomy (using structural MRI) and the functioning (using functional magnetic resonance imaging [fMRI], described below) of the brain with great precision. Structural MRI scans are based on the observation that the protons derived from hydrogen atoms, which are richly represented in the body because of its high water content, can be aligned by a magnetic field like small compass needles. When pulses are emitted at a particular frequency, the protons briefly switch their alignment and “relax” back into their original state at slightly different times in different types of tissue. The signals they emit are detected by the scanner and converted into highly precise images of the tissue. MRI methods have confirmed and extended findings from post mortem and CT scan studies—namely, that chronic use of alcohol results in brain shrinkage. This shrinkage is most marked in the frontal regions and especially in older alcoholics (Oscar-Berman 2000; Pfefferbaum et al. 1997; Sullivan 2000). Other brain regions, including portions of the limbic system and the cerebellum, also are vulnerable to shrinkage.

#### Imaging of Brain Function: Hemodynamic Methods

Hemodynamic methods create images by tracking changes in blood flow, blood volume, blood oxygenation, and energy metabolism that occur in the brain in response to neural activity. PET and SPECT are used to map increased energy consumption by the specific brain regions that are engaged as a patient performs a task. One example of this mapping involves glucose, the main energy source for the brain. When a dose of a radioactively labeled glucose (a form of glucose that is absorbed normally but cannot be fully metabolized, thus remaining “trapped” in a cell) is injected into the bloodstream of a patient performing a memory task, those brain areas that accumulate more glucose will be implicated in memory functions. Indeed, PET and SPECT studies have confirmed and extended earlier findings that the prefrontal regions are particularly susceptible to decreased metabolism in alcoholic patients (Berglund 1981; Gilman et al. 1990). It is important to keep in mind, however, that frontal brain systems are connected to other regions of the brain, and frontal abnormalities may therefore reflect pathology elsewhere (Moselhy et al. 2001).

Even though using low doses of radioactive substances that decay quickly minimizes the risks of radiation exposure, newer and safer methods have emerged, such as MRI methods. MRI is noninvasive, involves no radioactive risks, and provides both anatomical and functional information with high precision. The fMRI method is sensitive to metabolic changes in the parts of the brain that are activated during a particular task. A local increase in metabolic rate results in an increased delivery of blood and increased oxygenation of the region participating in a task. The blood oxygenation level–dependent (BOLD) effect is the basis of the fMRI signal. Like PET and SPECT, fMRI permits observing the brain “in action,” as a person performs cognitive tasks or experiences emotions.

In addition to obtaining structural and functional information about the brain, MRI methodology has been used for other specialized investigations of the effects of alcohol on the brain. For example, structural MRI can clearly delineate gray matter from white matter but cannot detect damage to individual nerve fibers forming the white matter. By tracking the diffusion of water molecules along neuronal fibers, an MRI technique known as diffusion tensor imaging (DTI) can provide information about orientations and integrity of nerve pathways, confirming earlier findings from post mortem studies which suggested that heavy drinking disrupts the microstructure of nerve fibers. Moreover, the findings correlate with behavioral tests of attention and memory (Pfefferbaum et al. 2000). These nerve pathways are critically important because thoughts and goal-oriented behavior depend on the concerted activity of many brain areas.

Another type of MRI application, magnetic resonance spectroscopy imaging (MRSI), provides information about the neurochemistry of the living brain. MRSI can evaluate neuronal health and degeneration and can detect the presence and distribution of alcohol, certain metabolites, and neurotransmitters.

#### Imaging of Brain Function: Electromagnetic Methods

In spite of their excellent spatial resolution—that is, the ability to show precisely where the activation changes are occurring in the brain—hemodynamic methods such as PET, SPECT, and fMRI have limitations in showing the time sequence of these changes. Activation maps can reveal brain areas involved in a particular task, but they cannot show exactly when these areas made their respective contributions. This is because they measure hemodynamic changes (blood flow and oxygenation), indicating the neuronal activation only indirectly and with a lag of more than a second. Yet, it is important to understand the order and timing of thoughts, feelings, and behaviors, as well as the contributions of different brain areas.

The only methods capable of online detection of the electrical currents in neuronal activity are electromagnetic methods such electroencephalography (EEG), event-related brain potentials (ERP),^4^ and magnetoencephalography (MEG). EEG reflects electrical activity measured by small electrodes attached to the scalp. Event-related potentials are obtained by averaging EEG voltage changes that are time-locked to the presentation of a stimulus such as a tone, image, or word. MEG uses sensors in a machine that resembles a large hair dryer to measure magnetic fields generated by brain electrical activity. These techniques are harmless and give us insight into the dynamic moment-to-moment changes in electrical activity of the brain. They show when the critical changes are occurring, but their spatial resolution is ambiguous and limited.

**Figure 3:**
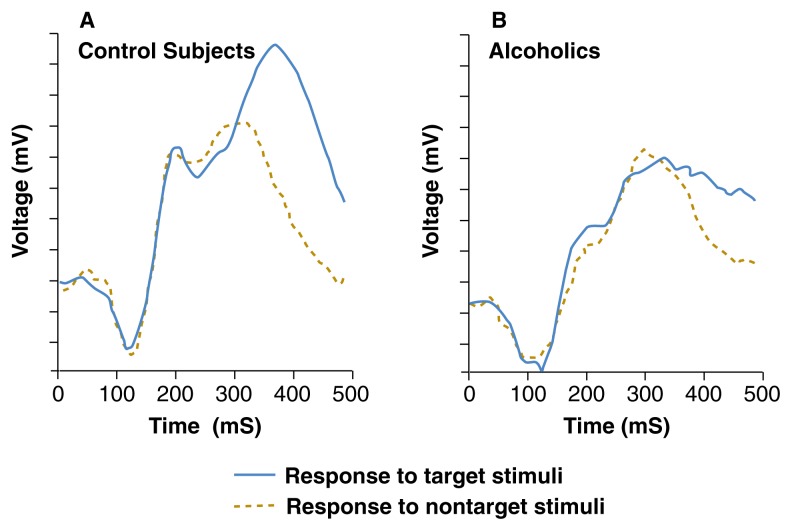
Brain electrical activity measured as event-related potentials (ERPs) in response to target stimuli (which require the subject to respond in some way) and nontarget stimuli (to be ignored by the subject). The brains of alcoholics are less responsive than the brains of nonalcoholic control subjects. The heights of the peaks are measured in terms of the strength of the electrical signal (volts) recorded from the scalp over time (in thousandths of a second, or mS). SOURCE: Porjesz and Begleiter 1995.

ERP and MEG have confirmed that alcohol exerts deleterious effects on multiple levels of the nervous system. These effects include impairment of the lower-level brain stem functions resulting in behavioral symptoms such as dizziness, involuntary eye movement (i.e., nystagmus), and insecure gait, as well as impairment of higher order functioning such as problem solving, memory, and emotion. ERP and MEG are remarkably sensitive to many alcohol-related phenomena and can detect changes in the brain that are associated with alcoholism, withdrawal, and abstinence. That is, these methods show different activity patterns between healthy and alcohol-dependent individuals, those in withdrawal, and those with a positive family history of alcoholism. As shown in figure 3, when brain electrical activity is measured in response to target stimuli (which require the subject to respond in some way) and nontarget stimuli (to be ignored by the subject), the brains of alcoholics are less responsive than the brains of nonalcoholic control subjects. Some of the ERP abnormalities observed in alcoholics do not change with abstinence, and similar abnormalities have been reported in patients who do not drink but come from families with a history of alcoholism. The possibility that such abnormalities may be genetic markers for the predisposition for alcoholism is under intensive scrutiny in studies combining genetic and electromagnetic measures in people with or without a family history of alcoholism (Porjesz and Begleiter 1998).

## Implications for Treatment

Because alcoholism is associated with diverse changes to the brain and behavior, clinicians must consider a variety of treatment methods to promote cessation of drinking and recovery of impaired functioning. With an optimal combination of neuropsychological observations and structural and functional brain imaging results, treatment professionals may be able to develop a number of predictors of abstinence and relapse outcomes, with the purpose of tailoring treatment methods to each individual patient. Neuroimaging methods have already provided significant insight into the nature of brain damage caused by heavy alcohol use, and the integration of results from different methods of neuroimaging will spur further advances in the diagnosis and treatment of alcoholism-related damage. Clinicians also can use brain imaging techniques to monitor the course of treatment because these techniques can reveal structural, functional, and biochemical changes in living patients across time as a result of abstinence, therapeutic interventions, withdrawal, or relapse. For example, functional imaging studies might be used to evaluate the effectiveness of drugs such as naltrexone on withdrawal-induced craving. (Naltrexone is an anticraving medicine that suppresses GABA activity.) Additionally, neuroimaging research already has shown that abstinence of less than a month can result in an increase in cerebral metabolism, particularly in the frontal lobes, and that continued abstinence can lead to at least partial reversal in loss of brain tissue (Sullivan 2000). Neuroimaging indicators also can be useful in prognosis, permitting identification and timely treatment of patients at high risk for relapse.

## Summary

Alcoholics are not all alike; they experience different subsets of symptoms, and the disease has different origins for different people. Therefore, to understand the effects of alcoholism, it is important to consider the influence of a wide range of variables. Researchers have not yet found conclusive evidence for the idea that any one variable can consistently and completely account for the brain deficits found in alcoholics. The most plausible conclusion is that neurobehavioral deficits in some alcoholics result from the combination of prolonged ingestion of alcohol, which impairs the way the brain normally works, and individual vulnerability to some forms of brain damage. Characterizing what makes alcoholics “vulnerable” remains the subject of active research.

In the search for answers, it is necessary to use as many kinds of tools as possible, keeping in mind that specific deficits may be observed only with certain methods, specific paradigms, and particular types of people with distinct risk factors. Neuroscience provides sensitive techniques for assessing changes in mental abilities and observing brain structure and function over time. When techniques are combined, it will be possible to identify the pattern, timing, and distribution of the brain regions and behaviors most affected by alcohol use and abuse. Electromagnetic methods (ERP and MEG) specify the timing of alcohol-induced abnormalities, but the underlying neural substrate (i.e., the anatomical distribution of the participating brain areas) cannot be unequivocally evaluated based on these methods alone. Conversely, the hemodynamic methods (fMRI, PET, and SPECT) have good spatial resolution but offer little information about the sequence of events. Drawing on the respective advantages of these complementary methods, an integrated multimodal approach can reveal where in the brain the critical changes are occurring, as well as the timing and sequence in which they happen (Dale and Halgren 2001). Such confluence of information can provide evidence linking structural damage, functional alterations, and the specific behavioral and neuropsychological effects of alcoholism. These measures also can determine the degree to which abstinence and treatment result in the reversal of atrophy and dysfunction.

## Footnotes

1 Alcohol dependence, also known as alcoholism, is characterized by a craving for alcohol, possible physical dependence on alcohol, an inability to control one’s drinking on any given occasion, and an increasing tolerance to alcohol’s effects (American Psychiatric Association [APA] 1994).

2 Some people may have better immunity than others to alcohol’s toxic effects.

3 The amount of alcohol needed to cause this effect depends on the person.

4 The ERP method is considered derived from electroencephalography.
